# A Review of Twenty Years of Research on the Regulation of Signaling Pathways by Natural Products in Breast Cancer

**DOI:** 10.3390/molecules27113412

**Published:** 2022-05-25

**Authors:** Muhammad Naeem, Muhammad Omer Iqbal, Humaira Khan, Muhammad Masood Ahmed, Muhammad Farooq, Muhammad Moeen Aadil, Mohamad Ikhwan Jamaludin, Abu Hazafa, Wan-Chi Tsai

**Affiliations:** 1 College of Life Science, Hebei Normal University, Shijiazhuang 050024, China; naeemsaleem413@gmail.com; 2 Shandong Provincial Key Laboratory of Glycoscience and Glycoengineering, School of Medicine and Pharmacy, Ocean University of China, Qingdao 266003, China; oiqbal133@gmail.com; 3 Royal Institute of Medical Sciences (RIMS), Multan 60000, Pakistan; 4 Department of Chemistry, University of Management and Technology, Lahore 54770, Pakistan; humairakhankhp@gmail.com; 5 Faculty of Pharmaceutical Sciences, Times Institute, Multan 60000, Pakistan; masoodkarni@yahoo.com; 6 College of Pharmaceutical Sciences, Zhejiang University, Hangzhou 310027, China; 7 Department of Zoology, Faculty of Science, Ghazi University, Dera Ghazi Khan 32200, Pakistan; mfarooq@gudgk.edu.pk (M.F.); moeen.adil@gmail.com (M.M.A.); 8 Bioinspired Device and Tissue Engineering Research Group, School of Biomedical Engineering and Health Sciences, Faculty of Engineering, Universiti Teknologi Malaysia, Johor Bahru 81310, Johor, Malaysia; mohamad.ikhwan@utm.my; 9 Department of Biochemistry, Faculty of Sciences, University of Agriculture Faisalabad, Faisalabad 38040, Pakistan; 10 Department of Medical Laboratory Science and Biotechnology, College of Health Sciences, Kaohsiung Medical University, Kaohsiung 807, Taiwan; 11 Department of Laboratory Medicine, Kaohsiung Medical University Hospital, Kaohsiung 807, Taiwan; 12 Department of Medical Research, Kaohsiung Medical University Hospital, Kaohsiung 807, Taiwan; 13 Research Center for Environmental Medicine, Kaohsiung Medical University, Kaohsiung 807, Taiwan

**Keywords:** breast cancer, natural products, transcription factors, signaling pathways, treatment, therapeutic agents, combination therapy

## Abstract

Breast cancer (BC) is the second leading cause of death among women, and it has become a global health issue due to the increasing number of cases. Different treatment options, including radiotherapy, surgery, chemotherapy and anti-estrogen therapy, aromatase inhibitors, anti-angiogenesis drugs, and anthracyclines, are available for BC treatment. However, due to its high occurrence and disease progression, effective therapeutic options for metastatic BC are still lacking. Considering this scenario, there is an urgent need for an effective therapeutic strategy to meet the current challenges of BC. Natural products have been screened as anticancer agents as they are cost-effective, possess low toxicity and fewer side effects, and are considered alternative therapeutic options for BC therapy. Natural products showed anticancer activities against BC through the inhibition of angiogenesis, cell migrations, proliferations, and tumor growth; cell cycle arrest by inducing apoptosis and cell death, the downstream regulation of signaling pathways (such as Notch, NF-κB, PI3K/Akt/mTOR, MAPK/ERK, and NFAT-MDM2), and the regulation of EMT processes. Natural products also acted synergistically to overcome the drug resistance issue, thus improving their efficacy as an emerging therapeutic option for BC therapy. This review focused on the emerging roles of novel natural products and derived bioactive compounds as therapeutic agents against BC. The present review also discussed the mechanism of action through signaling pathways and the synergistic approach of natural compounds to improve their efficacy. We discussed the recent in vivo and in vitro studies for exploring the overexpression of oncogenes in the case of BC and the current status of newly discovered natural products in clinical investigations.

## 1. Introduction

Cancer is considered as one of the most common causes of death worldwide, but no permanent treatment is available yet. Among cancers, breast cancer (BC) is the second leading cause of mortality among women, and it has become a global health challenge. It is estimated that about 7.8 million women were diagnosed in 2021 [1]. The global burden of BC is increasing every year in both developing and developed countries [2]. Many exogenous factors increased the risk and progression of BC associated with DNA damage, poor lifestyle, excessive alcohol consumption, nulliparity, diabetes, and estrogen replacement therapy [3,4,5]. About 5–10% of BC incidence occurs due to gene mutation events. BC progression is accelerated by elevated estrogens; leptin; and inflammatory mediators that promote BC cell proliferation, migration, and invasion [6].

Current treatment options for BC include radiotherapy, surgery, chemotherapy [7,8], anti-estrogen therapy, aromatase inhibitors, anti-angiogenesis drugs, and anthracyclines. However, long-term use of anti-estrogen therapy can cause serious health issues. Aromatase inhibitors increase the risk of cardiotoxicity. Anti-angiogenesis drugs also increased the risk of ischemic death. Anthracyclines can cause serious damage to the heart and nail tissues [9,10,11]. Moreover, these therapies are expensive and could cause toxic health effects. On the other hand, hormone therapy is another option at an early stage. However, patients are unable to show a response to hormone therapy. The excessive use of chemical drugs leads to increasing drug resistance and has become a major issue in the modern era. Nevertheless, chemical drugs are also causing serious health complications, such as liver cirrhosis, nausea, vomiting, and an increased risk of kidney failure (as toxic metabolites of drugs can cause serious cellular toxicities) [12]. The major challenges for BC treatment include chemoresistance, and there is a lack of effective therapeutic options for metastatic BC. Limited therapeutic options are currently available for triple-negative breast cancer (TNBC), such as chemotherapy. Due to poor responses to chemotherapy and aggressive behavior, there is an urgent need for a therapeutic strategy to meet the current challenges of TNBC. In order to overcome increasing drug resistance or side effects, natural products should be considered as viable therapeutic options with fewer side effects against TNBC. Therefore, there is a need for urgent BC treatment with no side effects [13,14].

Attention towards the safe use of natural products for BC treatment has increased over the past few years because they are cost-effective, low in toxicity, high in efficacy, and overcome drug resistance. Natural compounds are a rich source of bioactive compounds targeting tumor growth and cell invasion during BC progression [15,16]. The majority of therapeutic agents (>60%) for BC treatment are direct sources of natural products. In vitro and in vivo studies showed that consumption of an adequate amount of natural products obtained from plants, fruits, and vegetables is also helpful in the recurrence and reduction rate of BC, significantly increasing the survival rate among high-risk populations [17,18]. Various experimental studies showed that natural products showed inhibitory potentials for BC prevention through inhibiting angiogenesis, cell migrations, proliferations, and arresting the cell cycle by inducing apoptosis and cell death [19,20].

Recently, various natural products have been discovered and tested as anti-BC agents. These natural products are viridiflorol, verminoside, novel phloroglucinol derivatives, genistein, vulpinic acid, calcitrinone A, kaempferol, protopanaxadiol, thymoquinone, arctigenin, glycyrrhizin, 25-OCH3-PPD, oridonin, apigenin, wogonin, fisetin, curcumin, berberine, cimigenoside, and resveratrol [21,22,23,24,25,26,27,28,29,30]. These natural products are alkaloids, antioxidants, phenolic compounds, flavonoids, and polyphenols in nature, and are obtained from herbal products and vegetables. Consumption of these natural products in adequate amounts could target tumor cells, thus significantly reducing BC. Natural products also work synergistically to improve their efficacy against BC. Therefore, natural products could be used as a complementary therapy to improve the existing chemotherapy and reduce its side effects [31,32].

Natural products and derived bioactive compounds showed anticancer activities against BC by interacting with estrogen receptors, protein kinases, and the downstream regulation of signaling pathways (such as Notch, NF-κB- PI3K/Akt/mTOR, MAPK/ERK, and MDM2 pathways) by inducing apoptosis and cell death [23,33,34,35,36]. These pathways are misregulated following the invasion of BC and can be controlled through the action of natural products. Therefore, natural products have been used as an alternative therapeutic option for BC therapy. This review focuses on the emerging roles of novel natural products and derived bioactive compounds as therapeutic agents for BC therapy. The present review also discusses the mechanism of action through signaling pathways and the synergistic approach of natural compounds to improve efficacy. We have discussed the recent in vivo and in vitro studies for exploring the overexpression of oncogenes in the case of BC and the current status of newly discovered natural products in clinical investigations.

## 2. Pathogenesis of BC

**Figure 1:**
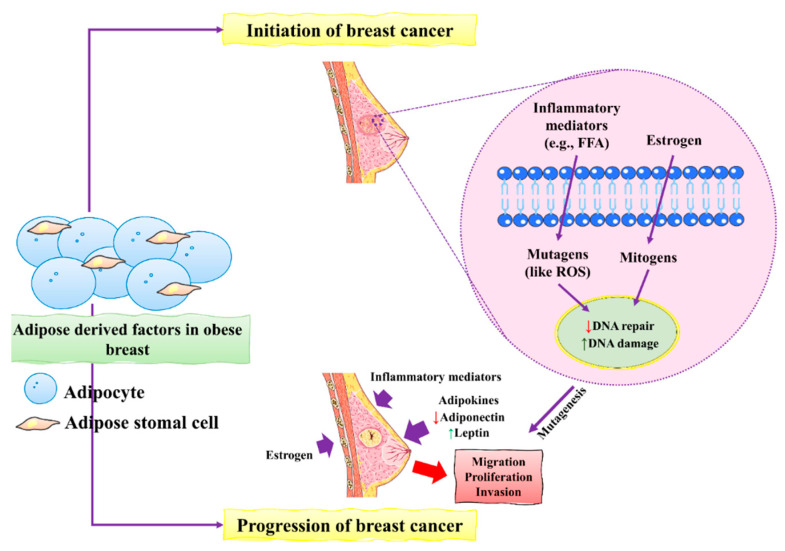
The initiation and progression of BC by obese breast adipose-derived factors. Factors released by obese breast adipose tissue may operate as mutagens, for example by activating intracellular reactive oxygen species (ROS), which can lead to DNA damage in normal breast epithelial cells and other inflammatory mediators. DNA damage may occur as a result of estrogen’s mitogenic actions, which can lead to replication stress and stress. Unresolved DNA damage, which is associated with mutagenesis and the onset of cancer, may result from increased DNA damage and possible estrogen-induced defective DNA repair. An obese breast adipose tissue microenvironment promotes BC proliferation, migration, and invasion by releasing inflammatory mediators, increasing leptin, decreasing adiponectin, and increasing estrogen levels. This figure is reproduced from Bhardwaj et al. [40] (Creative Commons Attribution License (CC BY 4.0)).

BC is the most common cause of malignant tumors worldwide. BC can occur from any mutational defect in breast ducts [37]. Generally, BC is categorized on the basis of estrogen receptors into ER-positive and ER-negative breast cancers [38]. Based on specific biomarkers, BC is further categorized into subtypes, such as luminal A, B, and basal-like. TNBC is the basal-like and most severe form (see Figure 1) [39].

**Figure 2:**
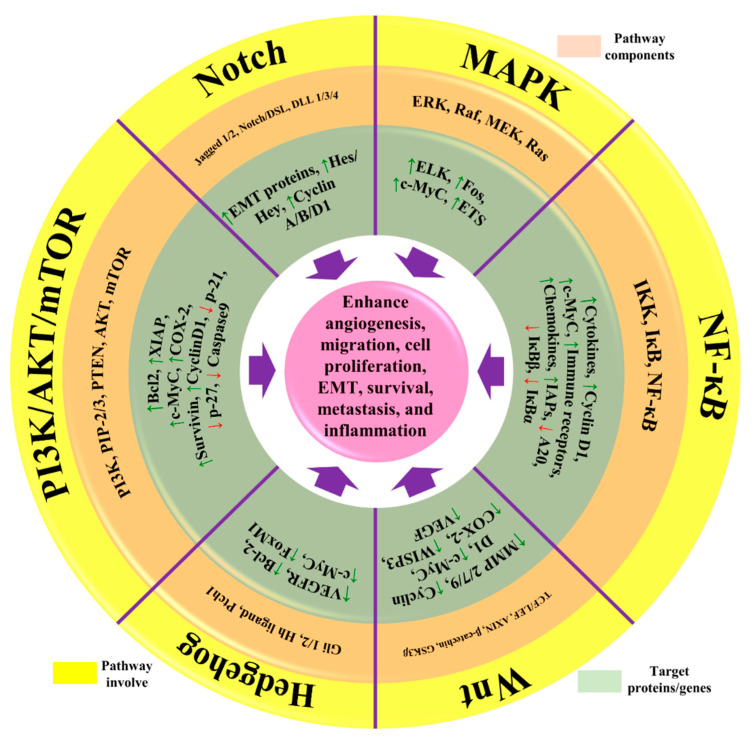
The representation of aberrant signaling pathways involved in BC. The green arrow represents the upregulation/activation, while the red arrow represents downregulation/inhibition. This figure is reproduced from Varghese et al. [44] (Creative Commons Attribution License (CC BY 4.0)).

Many environmental and genetic factors increase the risk of BC. These factors include increased damage to DNA, unusual hereditary mutations, exposure to estrogen, and a lifestyle that can increase the development of BC. Patients with a family history already in the malignant phase can also increase the chances of developing BC [11]. Most of the patients inherit their susceptible genes viz *p53*, *BRCA1*, and *BRCA2* [41]. Unusual mutation in CDH1 and overexpression of p53 can also increase the risk of developing BC [42]. RAS/MEK/ERK, PI3K/AKT, and RAS/MEK/ERK are the main pathways that help the normal cells to defend against cell death. Sometimes, mutational gene events in these pathways increase the chances of BC, as normal cells are incapable of committing cell suicide. For example, mutations in the PTEN gene activate the PI3K/AKT pathway, and cancerous cells are unable to go in commit suicide (see Figure 2) [43].

During BC development, epithelial cells change from neoplastic cells into cancerous cells. Adipose tissues mainly contribute to the initiation and progression of BC [40]. The main factors include the inflammatory mediators and mutagens in the form of estrogens that stimulate the production of ROS that ultimately causes severe damage to DNA in epithelial cells of the breast. Increased damage to DNA induced by estrogen causes severe defects and might lead to dysfunctional DNA repair. These unusual changes in DNA increased the risk of mutagenesis of BC. BC progression is accelerated by excess estrogens leptin and inflammatory mediators which promote BC cell proliferation, migration, and invasion [45].

In BC, chronic inflammation is mediated by tumor-infiltrating lymphocytes, cancer-associated fibroblasts, tumor cells, and tumor-associated macrophages. Inflammatory actions are triggered by either necrotic cells, such as damage-associated molecular patterns (DAMPS), or products released by microorganisms, such as pathogen-associated molecular patterns (PAMPS), in the case of breast cancer. As a result, innate and adaptive immune cells secreted the chemokines and cytokines that mediate inflammatory responses [46]. Angiogenesis is initiated by the activation of angiogenesis regulators IL-6, TNF-α, NF-κB, VEGF, Jun/Fos, and LPO. Different pathways are linked with chronic inflammation in BC, such as Notch, NF-κB, PI3K/Akt/mTOR, MAPK/ERK, and NFAT-MDM2 signaling pathways, as well as the regulation of EMT processes [47]. NF-κB is the most critical mediator during chronic inflammation in breast cancer. In NF-κB pathway, activation of different genes such as *IER3L*, *COX2*, *CXCL12*, and *CCND3* is a critical inflammatory mediator in patients with BC [48]. Various signaling pathways are activated during the inflammation of BC, which then leads to the activation of transcription factors, such as the signal transducer and activator of transcription signaling (STAT) and the activator protein 1 (AP-1) transcription factor [49].

Chronic inflammation in BC represented the seventh hallmark of cancer as compared to other malignancies. Various cellular events are major consequences of tumor progression, proliferation, and survival. Chronic inflammation indicates different events of BC, such as initiation and progression stages. Identifying different events would be helpful as an important strategy for controlling the BC among high-risk populations and prevention [50,51].

## 3. Downstream Regulation of Signaling Pathways by Natural Products

Natural products and derived bioactive compounds showed anticancer activities against BC by interacting with estrogen receptors through inhibiting tumor growth and protein kinases. This then helps to induce apoptosis and cell death using Bcl-2, caspases, p53, and p21; arrest the M/G2 phase of the cell cycle; increase the levels of CDK and cyclins, and regulate EMT processes. The downstream regulation of signaling pathways involves Notch, NF-κB, PI3K/Akt/mTOR, and MAPK/ERK pathways [24,52,53].

### 3.1. PI3K/Akt/mTOR Signaling Pathway

**Figure 3:**
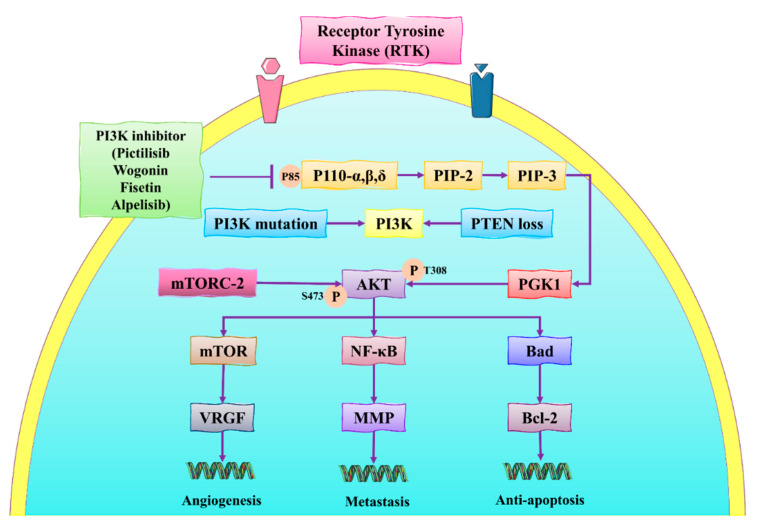
The schematic representation of the PI3K/Akt/mTOR signaling pathway in BC. VEGF: vascular endothelial growth factor; NF-κB: nuclear factor kappa-B; EGF(R): epidermal growth factor (receptor); mTOR: mammalian target of rapamycin; PDK1/2: 3-phosphoinositide-dependent kinase-1/2; PI3K: phosphatidylinositol 3-kinase; MMP: matrix metalloprotein; PIP-3: phosphatidylinositol (3,4,5)-trisphosphate; Bcl-2: B-cell lymphoma 2; mTORC-2: mTOR complex 2; VRGF: vascular endothelial growth factor; Bad: Bcl-2 antagonist of cell death. This figure is reproduced from Dong et al. [56] (Creative Commons Attribution License (CC BY 4.0)).

The PI3K/AKT/mTOR pathway is one of the most important signaling pathways activated in BC and participates in cellular activities, such as cell proliferation, invasion, and cell migration [52]. This pathway also suppresses apoptosis and thus increases the growth of abnormal breast tissues. It is also essential to overcome the increasing resistance to drugs used against BC. Suppressing the activation of different genes involved in this pathway is helpful in developing a novel strategy for BC prevention [53]. Activation of PI3K promotes the activation and phosphorylation of AKT, which is the main constituent in tumorigenesis. On the other hand, mTOR regulates cell proliferation and induces apoptosis in BC cells under exposure to chemotherapy. One of the potential strategies used to prevent BC is suppressing or downregulating the PI3K/Akt/mTOR pathway through natural products (see Figure 3) [54,55].

Mutations in the *PIK3CA* gene lead to an increase in the risk of BC. The mutated *PIK3CA* gene activates the PIK3, initiating the tumor growth in breast tissues that comprised p85 and p110 [57,58]. PIK3 interacts with the VEGFR and promotes angiogenesis. PIK3 converts the phosphatidylinositol 3,4-bisphosphate (PIP2) into 3,4,5-triphosphate (PIP3), interacts with phosphoinositide-dependent kinase-1 (PDK1), and catalyzes the phosphorylation of AKT at Thr 308. AKT also helps in the phosphorylation of mTOR and MPP which activates NF-κB, thus promoting metastasis [59,60]. Hong et al. [61] conducted in vitro and in vivo studies for investigating the role of ginsenoside Rk1 against BC cells (MDA-MB-231). They demonstrated that ginsenoside Rk1 inhibited the PI3K/Akt pathway, stimulated ROS production, increased the expression of Bax, reduced Bcl-2 levels, and activated the release of cytochrome-c from the mitochondrial membrane, and activated caspase 3/8. These molecular events induced apoptosis in MDA-MB-231. Another recent in vivo study conducted by Liu et al. [62] showed that the administration of ginsenoside Rg5 (20 mg/kg) for 30 days significantly inhibited the PI3K/Akt pathway during BC treatment in a BALB-c nude mice model. Therefore, these pathways can be regulated through the action of natural products.

Voacamine (VOA) is a bis-indole alkaloid that is isolated from *V. africana* and showed anticancer activities against BC [63]. Zuo et al. [34] conducted an in vitro study to investigate the role of VOA in BC treatment by downregulating the PI3K/Akt/mTOR. VOA showed its usefulness against MCF-7 and 4T1 cells with an IC_50_ value of 1.48 μM. VOA also significantly inhibited the phosphorylated AKT and mTOR in BC cells and also decreased the expression of CDK2 and cyclin A/E. It also induced apoptosis and cell death in MCF-7 and 4T1 cells by arresting the S phase of the cell cycle. VOA induced mitochondrial apoptosis by inhibiting the activity of MMP and increased the level of cytochrome-c in the cytoplasm. Cytochrome-c interacts with protease-activating factors that cleave the assembly of caspase 8/9 and stimulate their activation. It also induced apoptosis which underlies the mitochondrial apoptotic pathway in BC cells [64].

Fisetin is a flavonoid-based natural product that showed anticancer potential against BC. It is found in cucumber, apple, strawberry, and onion. It induced apoptosis in BC cells, inhibiting the cell migrations and suppressing the tumor growth. It also inhibited the expression of Bcl-2 in BC cell lines (MDA-MB-231) [22,65]. Sun et al. [66] conducted in vitro and in vivo (BALB/c mice) studies to investigate fisetin’s role in BC. They found that fisetin induced apoptosis in MCF-7, 4T1, and MDA-MB-231 at 40 and 80 μM. They also found that fisetin acted as an inhibitor of PI3K/Akt/mTOR signaling and inhibited the proliferation and dysregulation of this signaling pathway. The low availability of fisetin in vivo studies limits their use in clinical investigations [67].

Wogonin (WG) is a natural product with a flavonoid nature and is found in the root of *S. baicalensis*. WG is also used for BC therapy, as it showed inhibitory potentials against BC cell lines (MCF-7 and MDA-MB-231) [68]. It also plays an important role in the downregulation of PI3K/Akt/mTOR pathway. Several studies showed that WG is also used in BC to overcome drug resistance [69]. Zhao et al. [70] conducted in vitro and in vivo studies and showed that WG can have inhibitory effects on MCF-7/MDA-MB-231 and the chicken chorioallantoic membrane (CAM) model, respectively, at 20 and 40 μM. They also found that WG acted as an inhibitor of PI3K/Akt/mTOR signaling and showed inhibition of the proliferation and downregulation of this signaling. 1,3,4,9-tetrahydropyran [3,4-b]-indoles showed anticancer activity against MDA-MB-231 cells with IC_50_ (2.29 μM). It also induced apoptosis and cell death of MDA-MB-231 cells by arresting the G0/G1 phase of the cell cycle. These recently discovered natural products are involved in downregulating the PI3K/Akt/mTOR pathway, thus acting as promising candidates for BC treatment.

### 3.2. NF-κB Signaling Pathway

Nuclear factor-kappa B (NF-κB) is one of the most important transcription factors that are activated during inflammation, tumor growth, and proliferation of BC cells. Activation of NF-κB in BC is crucial, and its regulation can be a therapeutic strategy for BC therapy [71]. Natural compounds showed interaction with NF-κB and blocked their activities through dephosphorylation that consecutively deactivates p50. IκB can be activated through the IκB kinase which promotes its phosphorylation and is helpful for the activation of p50, initiating transcription by entering into the nucleus via nuclear pores. This activation stimulates NF-κB for the genetic expression of sacral genes that cause inflammatory responses [71,72].

**Figure 4:**
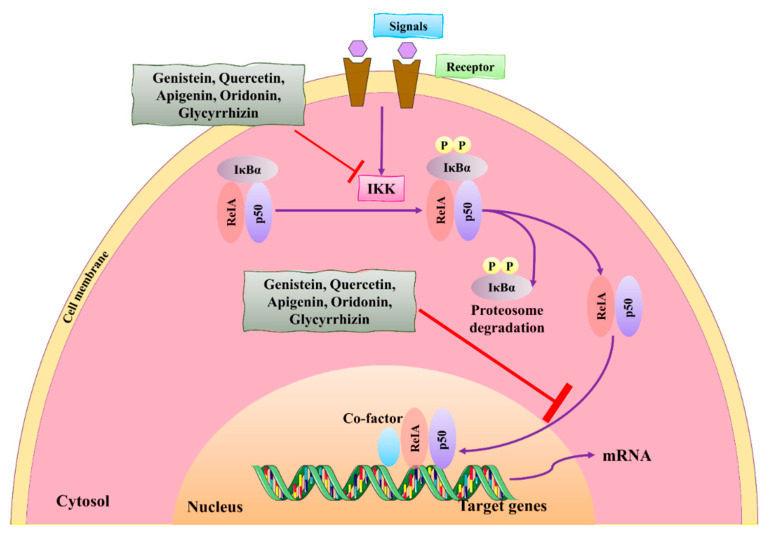
The role of natural compounds in the treatment of BC by regulating the NF-κB signaling pathway and by suppressing the transcription of NF-κB-targeted genes. IKK: IκB kinase. This figure is reproduced from Ganesan et al. [76] after gaining permission from Elsevier (license no. 5310141484610).

Natural compounds showed anticancer and antitumor activities by downregulating and suppressing the NF-κB pathway. These compounds, genistein and quercetin, can inhibit the phosphorylation of IκBα in MCF-7 HER2 cell lines, thus playing a significant role in the regulation of IκBα to the p50 (see Figure 4) [73]. These compounds also inhibited the NF-κB pathway by blocking the phosphorylation of p65 in the nucleus, thus inhibiting the nuclear translocation [74]. These events also inhibited the functions of NF-κB-targeted genes. Another study found that the application of genistein could control the activation of NF-κB and showed maximum potential at an IC_50_ value of 20 µM against MDA-MB-231 cells [74,75].

Apigenin (AP) is another natural product with a flavone nature isolated from *A. cepa* and *C. sinensis*. It exhibited anticancer activity against BC cell lines (MDA-MB-231) [21]. It influenced the NF-κB pathway by suppressing the COX-2 and downregulating the gene expression of NF-κB. It also inhibited cell proliferation and migration by arresting the cell cycle at the G2/M phase. It also suppresses the cyclin A, B, and CDK1 which can control the G2/M phase. Bauer et al. [77] investigated the anticancer activity of AP against BC cells (MDA-MB-231) and found that it influenced the NF-κB pathway by suppressing the VEGF through deactivating progesterone receptors in BC cells.

Ginsenosides and their derivatives are anticancer-based natural products extracted from *Panax ginseng* with great potential against BC. Kim et al. [78] conducted in vitro and in vivo studies to investigate the role of ginsenoside Rg3 against BC cells (MDA-MB-231). They found that ginsenoside Rg3 inhibited the NF-κB pathway by inhibiting the phosphorylated AKT and ERK, and induced apoptosis and cell death in MDA-MB-231.

Glycyrrhizin (GLA) is a terpenoid-based natural product isolated from *G. glabra*. It showed anticancer activity against MDA-MB-231 by inhibiting invasion and cell proliferation, and also inhibited the E-cadherin [25]. These novel natural products are involved in downregulating the NF-κB pathway, thus acting as promising candidates for BC treatment.

### 3.3. MAPK/ERK Signaling Pathway

**Figure 5:**
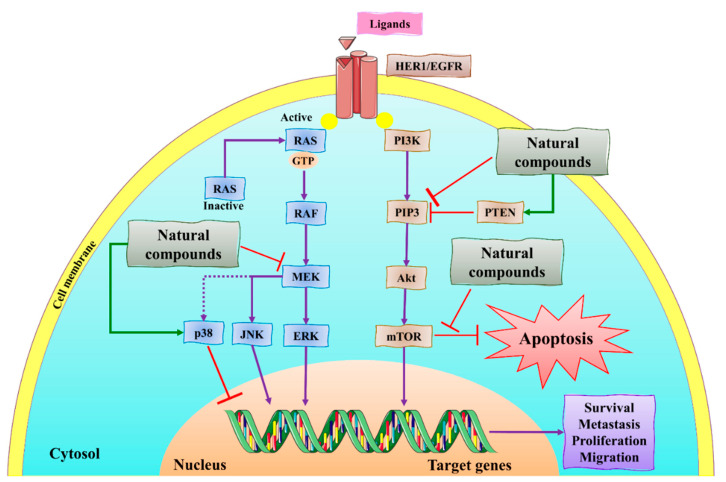
The schematic representation of the mechanism of action of natural compounds on MAPK/ERK and PI3K/Akt/mTOR signaling pathways can inhibit migration, survival, cell proliferation, and metastasis. PTEN: phosphatase and tensin homolog; MEK: mitogen-activated protein kinase; JNK: c-Jun N-terminal kinase; Akt: protein kinase B; ERK: extracellular signal-regulated kinase; mTOR: mammalian target of rapamycin; PIP-3: phosphatidylinositol (3,4,5)-trisphosphate. This figure is reproduced from Ganesan et al. [76] after gaining permission from Elsevier (License No. 5310141484610).

The MAPK/ERK signaling pathway is another molecular cascade used for the activation of several genes in BC. These genes are *ERK 1/2* and *JNK 1/2*, which play a significant role in cell proliferation by activating transcription factors. The inactivation of genes through natural products can inhibit tumor growth and invasion in carcinogenic mechanisms in this signaling pathway. Natural compounds showed their ability to interact with MAPK/ERK transcription factors, thus downregulating the signaling pathway (see Figure 5). Arctigenin (ATG) showed maximum potential at 200 μM and was directly involved in suppressing the MAPK pathway by inhibiting the phosphorylation of JNK and ERK in MCF-7 and MDA-MB-231 cells [24]. Other studies showed that delphinidin, a natural compound of anticancer activity, was also involved in suppressing the MAPK pathway by inhibiting the phosphorylation of *JNK 1/2* and ERK in MDA468 and MCF-7 cells [79]. Thymoquinone also downregulated the MAPK pathway by inhibiting the activation of p38 and JNK [80]. Protopanaxadiol (PPD) targeted BC cell lines by suppressing the MAPK pathway through deactivation of *ERK1/2*, p38, and JNK, and showed maximum activity against MDA-MB-231 below 20 μM [81].

Kaempferol exhibited anticancer activities against BC (MDA-MB-453) by arresting the G2/M phase of the cell cycle [11]. It bound with CDK1 and blocked their activities. Zhu et al. [82] conducted an in vitro (BT474 and MDA-MB-231) study to investigate the role of kaempferol in treating BC and found that the number of cancerous cells decreased from 85.2% to 50.32% in the G1 phase of the cell cycle. These studies showed that kaempferol can significantly inhibit the BC cells by blocking the critical phases of the cell cycle. It induces apoptosis and ultimately leads to cell death [82]. The growth and proliferation of BC cells can depend on glucose utilization. Inhibiting glucose decreased the survival rate of cancerous cells, thus making it a promising therapy for early-stage BC treatments [83]. Azevedo et al. [84] conducted in vitro and in vivo studies to investigate the uptake of glucose and the absorption of lactate with MCF-7 BC cells. They found that kaempferol inhibited the proliferation of tumor cells, thus minimizing the utilization of glucose by MCF-7 BC cells. On the other hand, kaempferol also decreased the absorption of lactate to MCF-7 BC cells that cannot survive, thus leading to cell death.

Hung et al. [85] investigated the anticancer role of kaempferol and found that binding with estradiol can degrade ERα and inhibit the proliferation of MCF-7 cells. Kim et al. [33] also conducted an in vivo study in a mice model (BALB/c nu/nu) to discover the anticancer potentials against MCF-7 BC cells. They also found that the binding of kaempferol with triclosan causes the suppression of the ER signaling pathway. These events activate the RAS protein that induces cell proliferation and causes cell death in BC cells. Thus, natural compounds showed inhibitory actions against different breast cell lines by inhibiting the expression of genes involved in the MAPK/ERK pathway.

### 3.4. Notch Signaling Pathway

**Figure 6:**
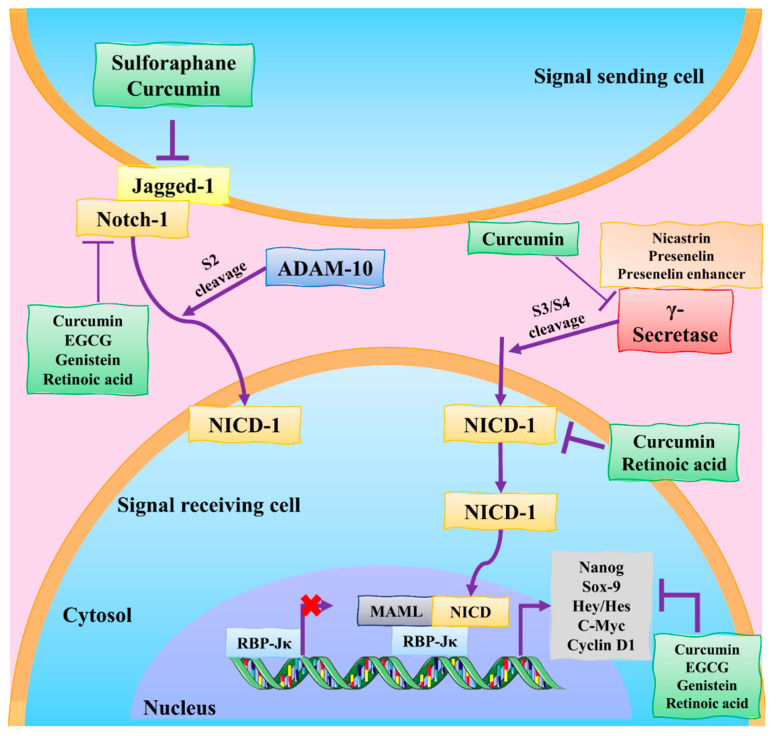
Natural products regulate the modulation of the Notch signaling pathway in BC. The Notch receptor is cleaved by Notch ligands, including Jagged-1. ADAM-10 initially cleaves the Notch extracellular domain; the gamma–secretase complex then cleaves the Notch intracellular domain (NICD). NICD moves into the nucleus and initiates transcription. MAML: mastermind-like protein; EGCG: epigallocatechin-3-gallate; RBP-Jκ: recombinant signal binding protein for immunoglobulin kappa J region; DATS: diallyl trisulfide; ADAM-10: a disintegrin and metalloproteinase domain-containing protein 10; NICD: Notch receptor intracellular domain. This figure is reproduced from Kiesel et al. [87] (Creative Commons Attribution License (CC BY 4.0)).

Various anti-apoptotic proteins (Bcl-2) are involved in regulating the Notch pathway and thus play a significant role in maintaining mitochondrial permeability [86]. Gamma secretase is the critical enzyme involved in the Notch intracellular domain (NICD) transcription via the Notch pathway (see Figure 6). NICD moves from the cytoplasm to the nucleus and binds with it to regulate transcriptional complexes containing DNA-binding protein CBF1/RBPjk/Su(H)/Lag1 (CSL) which downregulate the Notch pathway [87]. Cimigenoside, a novel natural compound isolated from *C. dahurica*, also inhibited the release of gamma–secretase from the transmembrane region by inhibiting their catalytic core PSEN-1, ultimately inhibiting the binding of NICD to CSL, and thus marinating the regulation of the Notch pathway. Cimigenoside showed its usefulness by inducing apoptosis and inhibiting Bcl-2, which ultimately then inhibited the Notch pathway [23,88,89].

Unusual abnormalities in the form of malignant tumors in the Notch signaling pathway lead to a variety of cancers, such as BC. Cimigenoside inhibited the Notch signaling pathway by interrupting various proteins. It tightly binds with the PSEN-1 and inhibits their activity [90,91]. Cimigenoside also degraded NICD in the nuclear region. It does not affect the expression of PSEN-1 in the cytoplasm. Thus, cimigenoside is directly involved in the inactivation of PSEN-1 and decreases the level of NICD in the nuclear region. Bcl-2 inhibited apoptosis, thus regulating the Notch signaling pathway. Cimigenoside also inhibited the activity, or interrupted the balance, of Bax and Bcl-2 and induced apoptosis in BC cells [92].

Jia et al. [23] performed in vivo (Balb/C Nude Crlj mice) and in vitro (MDA-MB-231 and MCF-7) studies on *C. dahurica* isolated to investigate the role of cimigenoside as an anticancer agent in BC treatment. The isolated cimigenoside showed maximum anticancer activity against BC cell lines (MDA-MB-231 and MCF-7) with IC_50_ (12.6 ± 1.47, 15.6 ± 2.47 μM). Cimigenoside induced apoptosis in BC cells by arresting the G2/M phase of the cell cycle. Another in vitro study screened the isolated compound, β-d-allopyranosyl-3-methoxyphenyl, from *Cimicifuga dahurica*. This isolated compound exhibited excellent anticancer potentials against BC cell lines (MCF-7 cells) with an IC_50_ value of 30 μM. They showed that this compound induced apoptosis by decreasing the expression of Bcl-2 and Bcl-XL which acted as anti-apoptotic proteins with anti-proliferation effects by inhibiting c-Myc and cyclin D1, and also arrested the cell cycle at G0/G1-S [90]. Oridonin is a diterpenoid-based natural product that was isolated from *R. rubescens*. By blocking the Notch signaling system and inhibiting angiogenesis and EMT linked to VEGF-A, this drug may be effective in the fight against breast cancer development and spread [88]. Xia et al. [89] conducted an in vivo experiment in BALB/C athymic nude mice and reported that oridonin successfully induced apoptosis in human BC cells. They also found that Notch 1−4 protein expression was also lowered by oridonin therapy, which hindered cancer cell migration and invasion. Thus, natural compounds showed inhibitory properties against different BC cell lines for regulating the Notch pathway.

### 3.5. MDM2 Signaling Pathway

MDM2 signaling pathway is another pathway activated during BC progression. p53 is a tumor suppressor protein that plays a significant role in the regulation of the cell cycle. It is encoded by the *TPp53* gene. It is important for various cellular events occurring during the cell cycle, such as apoptosis. Overexpression of the *p53* gene leads to an increase in the risk of BC. Its expression can be controlled by murine double minute 2 (MDM2) through a negative feedback mechanism in two ways [93,94]. In the case of non-stress environments, MDM2 tightly binds to the p53 via the transactivation domain. After binding with p53, it starts gradual degradation in the presence of ubiquitination. Without ubiquitination, degradation of p53 becomes slow. Under stress environments, in case of damage to DNA, a complex of MDM2-p53 has stabilized as stress facilities this process. Any defect in MDM2 can decrease the binding and degradation activities, thus losing negative feedback [36,95,96].

25-OCH3-PPD is one of the most active ginsenosides isolated from *P. notoginseng* and acts as a therapeutic agent for treating BC. Wang et al. [36] conducted in vitro and in vivo studies to demonstrate its anticancer activities against MDA-MB-231 and nu/nu mice, respectively. They found that 25-OCH3-PPD acted as an inhibitor of MDM2 at the transcriptional level. They observed that 25-OCH3-PPD was also involved in arresting the G1 phase of the cell cycle and induced apoptosis in BC cells. 25-OCH3-PPD also plays an essential role in inhibiting tumor growth, cell invasion, and the MDM2 pathway in BC. 25-OCH3-PPD could serve as a potential anticancer agent for BC therapy. Ginsenosides, a natural product, targeted the tumor cells and induced apoptosis and cell differentiation in BC. It also acts as an inhibitor of MDM2, which might be a potential target in BC therapy. These novel natural products are involved in downregulating the MDM2 pathway, thus acting as promising candidates for BC treatment [97].

**Figure 7:**
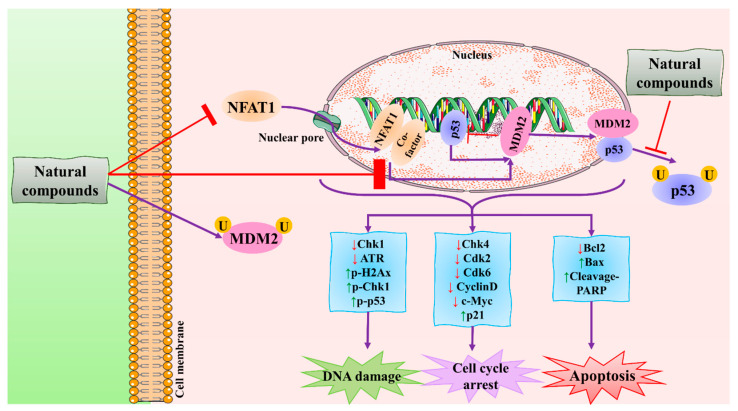
The schematic representation of the mechanism of action of natural compounds against BC using the MDM2 signaling pathway. Natural compounds induce apoptosis by inhibiting the NFAT1-MDM2 signaling pathway to reduce cancer. NFAT1: nuclear factor of activated T cells 1; MDM2: mouse double minute 2 homolog; CDK: cyclin-dependent kinase; Chk: checkpoint kinase; Bcl-2: B-cell lymphoma 2; Bax: BCL2-associated X protein; PARP: poly (ADP-ribose) polymerase. This figure is reproduced from Qin et al. [95] (Creative Commons Attribution License (CC BY 4.0)).

Lineariifolianoid A (Lin A) is another sesquiterpenoid-based natural product isolated from *I. lineariifolia* which could be used against BC [98,99]. The nuclear factor of activated T-cells (NFAT) and MDM2 are oncogenes that play an important role in BC cell proliferation, invasion, and migration. Both oncogenes are overexpressed in the case of BC. Several therapeutic agents have been used as an inhibitor of NFAT and MDM2. One of the important targets of NFAT and MDM2 is Lin A [100]. Qin et al. [95] conducted in an vitro study to investigate Lin A’s role in inhibiting the NFAT-MDM2 pathway. They found that Lin A arrested the cell cycle at the G2/M phase and inhibited cell invasion and cell proliferation in BC cells. They also observed that Lin A induced apoptosis at a higher concentration of 50% in BC cells (MCF7 and MDA-MB-231 with an IC_50_ value of 4.5 ± 0.3 and 7.8 ± 0.6 µM, respectively. Therefore, Lin A acted as a novel inhibitor of NFAT-MDM2 pathways and can be used as a therapeutic agent for treating BC therapy (see Figure 7). Detailed information about the individual and synergistic natural products with better anti-cancer activities is presented in Table 1 with a proper mechanism of action.

## 4. Synergistic Approach of Natural Products against BC

Several chemotherapeutic drugs have been used to treat BC. The development of chemotherapeutic drugs has increased the resistance rate in BC cells and remained a challenge for breast therapy [109,110]. Drug resistance and tumor growth are critical factors for increased mortality risk among women. The prolonged use of a single drug is not effective for the treatment of BC [111]. Therefore, the clinical use of these drugs has become critical for BC. Therefore, urgent therapy with a safe mode on breast tissues can overcome this issue.

For the complete eradication of BC, combinations of natural products with drugs are an effective strategy to overcome the side effects and minimize the risk of recurrence of BC cases [112]. Therefore, combining the effects of natural products is an important therapeutic strategy for overcoming drug resistance and controlling the BC in high-risk populations.

### 4.1. Synergistic Effects of Curcumin and Berberine

**Figure 8:**
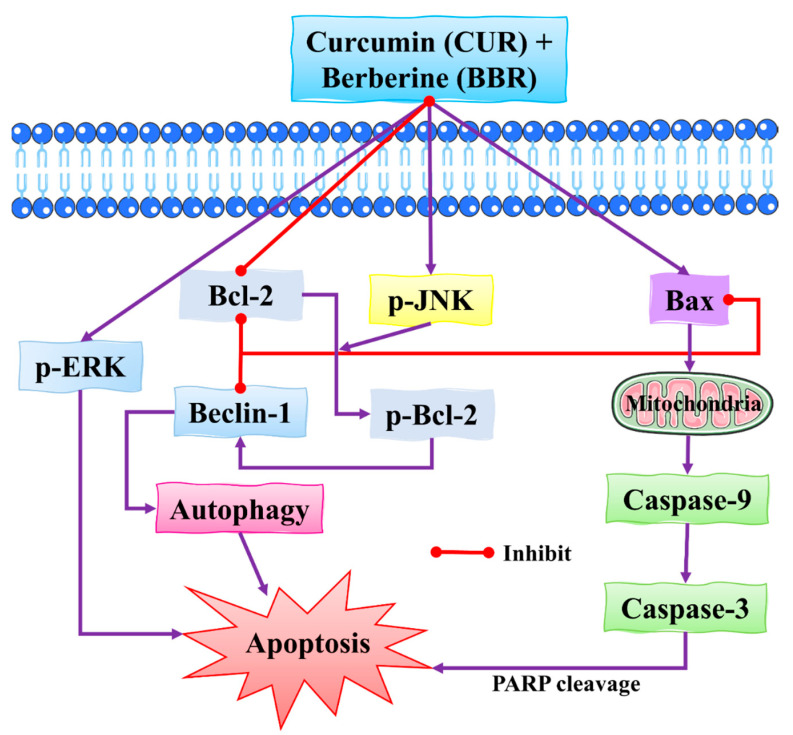
The synergistic effect of CUR and BBR against BC cells by inducing apoptosis. Apoptosis in BC cells is induced by a combination of CUR and BBR, mediated through the activation of the ERK signaling pathway. These two compounds work together to enhance JNK activation, phosphorylation of Bcl-2, and dissociation of the Beclin1/Bcl-2 complex in BC cells, eventually resulting in autophagic cell death. Bcl-2: B-cell lymphoma 2; Bax: BCL2-associated X protein; PARP: poly (ADP-ribose) polymerase; JNK: c-Jun N-terminal kinase; ERK: extracellular signal-regulated kinase. This figure is reproduced from Wang et al. [104] (Creative Commons Attribution License (CC BY 4.0)).

Some natural products have been used as combinations with synergistic effects to improve their efficacy against BC. For example, curcumin (CUR) and berberine (BBR) have been used as potential sources of natural products to treat BC. CUR and BBR were isolated from the root of *Curcuma longa* and *Rhizoma coptidis* respectively in the search for secondary metabolites with anticancer, antitumor, and anti-inflammatory properties [113,114]. CUR and BBR are of great interest in clinical investigations because of their low toxicity. Recently, the synergistic effects of CUR and BBR have shown excellent anticancer properties against BC (see Figure 8) [115,116].

CUR is a diarylheptanoid-based natural compound, and its chemical structure reveals the presence of carbonyl and phenolic groups. CUR is mainly composed of turmeric compounds (2–8%) and is an excellent source of yellow turmeric [117]. It is also used in TNBC as it interferes with the EMT process and inhibits BC cell migration [118]. Its anticancer activities induced apoptosis and cell death in BC cells in order to prolong the survival of cells [119]. BBR is an isoquinoline alkaloid that exhibits anticancer activities and induced apoptosis and cell death in BC cells. Its anticancer activities are reflected by its binding ability with DNA topoisomerase, as well as the ability to induce apoptosis in BC cells [120]. Genus *Berberis* is an excellent source of BBR [121]. It is widely used for the treatment of TNBC due to inhibition and migration of BC cell MDA-MB-231. BBR is also used as a potential agent for breast therapy due to its ability to reverse MDR. Therefore, combinations of natural products showed robust effects in reducing invasion, migration, and the EMT process [122,123].

Wang et al. [104] conducted an in vitro study to investigate the combined effects of CUR and BBR on the MCF-7 and MDA-MB-231 BC cells. The synergistic effects of both natural compounds induced apoptosis and cell death via the activation of the ERK and JNK signaling pathways which regulated the phosphorylation of JNK and decreased the phosphorylation of Bcl-2. The combined effects of CUR and BBR played a significant role in preventing BCs; thus, they can be used for BC therapy.

CUR and BBR are widely used for the treatment of BC. They exhibit a polyphenol nature, and affect various cellular processes, including EMT, as in the case of BC. Thus, the synergistic effects of these natural compounds are helpful in combating BC as compared to traditional chemotherapies [122,124]. Another recent in vitro study conducted by Kashyap et al. [105] demonstrated that combinations of CUR + BBR impaired the EMT process, thus showing effects against BC cell lines (MDA-MB-231 and MDA-MB-468) with *p* ≤ 0.010. Thus, synergistic combinations of CUR and BBR could be used as potential therapeutic agents for BC therapy.

### 4.2. Synergistic Effects of Baicalin and Methylglyoxal with 5-Fluorouracil

**Figure 9:**
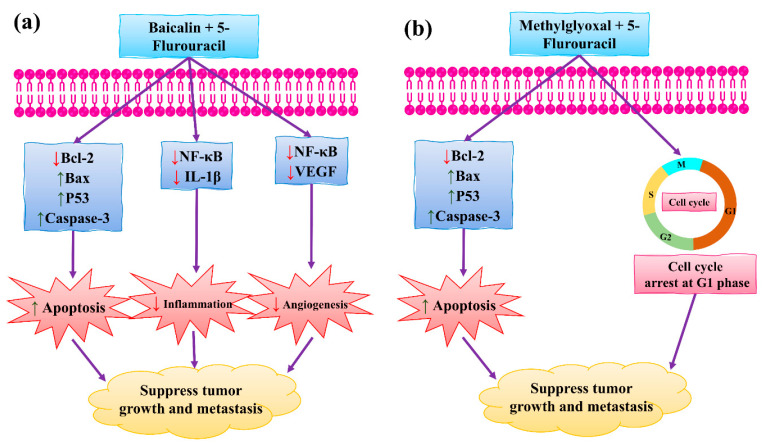
The synergistic effect of (**a**) BA and 5-FU, and (**b**) MG and 5-FU in BC therapy. BCL2: B-cell lymphoma two protein; NF-κB: nuclear factor kappa B; Bax: Bcl-2-associated X protein; p53: inducible gene 3; IL-1β: interleukin 1 beta; VEGF: vascular endothelial growth factor. This figure is reproduced from Shehatta et al. [106] (Attribution Non-Commercial No. Derivatives 4.0 International (CC BY-NC-ND 4.0)).

Baicalin (BA) is a flavonoid-based natural compound isolated from *Scutellaria baicalensis* that exhibited excellent anticancer, anti-proliferative, and antioxidant properties [125]. BA is non-toxic for humans. It regulates the normal development of the breast by inhibiting the proliferation and invasion of cancer cells, thus triggering apoptosis which ultimately leads to cell death (see Figure 9). Chung et al. [26] have demonstrated that BA can suppress the NF-κB pathway in the development of human breast epithelial cells. Other studies showed that BA significantly prevents BC, including inhibited tumor growth, angiogenesis, fibrosis, invasion, and apoptosis [126]. Methylglyoxal (MG) showed anticancer activity against BC by inhibiting ATP depletion, and by suppressing glycolysis and mitochondrial respiration [127,128]. 5-fluorouracil (5-FU) has also been used as a chemotherapeutic drug to treat BC. It binds with DNA by blocking the activity of thymidylate synthase. It is also involved in targeting invasion cells and in inhibiting their proliferation. Other roles include the activation of p53 by inducing apoptosis [129,130]. It is known that toxic metabolites released from 5-FU metabolism also block DNA synthesis [131]. The side effects of 5-FU have been reduced through combinations with natural products. For example, 5-FU itself is a toxic agent, but combinations positively impacted BC prevention [106]. 5-FU is widely used for the treatment of BC. It inhibited the invasion and progression of cancer cells by inducing apoptosis, ultimately leading to cell death. Clinical trials in combination therapy with other natural products are needed to overcome chemoresistance in BC [131].

Shehatta et al. [106] demonstrated the combined effect of 5-FU and BA on in vivo animal models (Swiss albino mice) and revealed that they significantly suppress the NF-κB signaling pathway. The surviving (IL-1β, Bcl-2, and VEGF) and upregulating (p53, caspase-3, and Bax) genes were involved in this suppression. Another study conducted by Miao et al. [132] demonstrated that the combined effect of 5-FU and BA reduced inflammation by inhibiting VEGF, IL-1β, and NF-κB. They found that it induced apoptosis by caspase-3 and Bax. In view of the above, BA acts as a promising candidate for improving BC therapy.

### 4.3. Synergistic Effects of Resveratrol and Salinomycin

**Figure 10:**
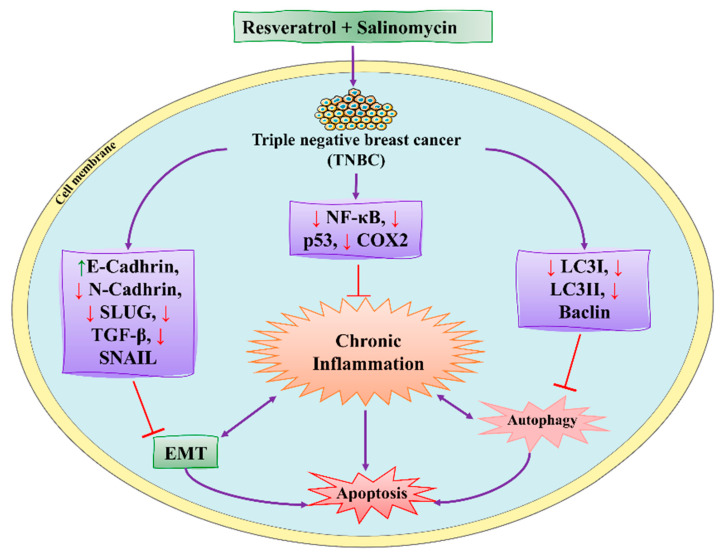
RSVL and SAL synergistic effect against BC cells by inducing apoptosis. NF-κB: nuclear factor kappa B; p53: inducible gene 3; TGF-β: transforming growth factor-beta; EMT: epithelial to mesenchymal transition; COX2: cyclooxygenase-2. This figure is reproduced from Rai et al. [107].

Resveratrol (RSVL) is a polyphenolic compound and showed anticancer activity against BC cells (MDAMB-231 and MCF-7). It is obtained from dietary sources, such as peanuts, grapes, and berries. RSVL activities include arresting the S1 phase of the cell cycle and inducing apoptosis in BC cells [133]. It acts as an antioxidant by preventing DNA damage and suppressing tumor growth (see Figure 10) [108]. RSVL also inhibited ATP in MCF-7. Thus, RSVL, as a natural product, decreases MDAMB-231 and MCF-7 cells [134]. Salinomycin (SAL) is a potent antibiotic used for the treatment of BC. It is derived from *Streptomyces albus* and was useful for inhibition, cell proliferation, and the suppression of tumor growth [135]. SAL also induced apoptosis and cell death in metastatic BC cells by promoting DNA damage and elevating ROS production. Some in vivo studies showed that SAL significantly suppressed tumor growth [136].

RSVL and SAL acted synergistically to improve the efficacy of BC therapy. Rai et al. [107] investigated the role of RSVL and SAL in vitro and in vivo studies against MDA-MB-231 BC cells and Swiss albino mice, respectively. They found that synergistic combinations of RSVL and SAL inhibited the epithelial–mesenchymal transition and suppressed p53, COX-2, and Beclin. They also found that RSVL and SAL induced apoptosis in TNBC. Dewangan et al. [137] investigated the role of SAL and RSVL against BC. They found that this combination induced apoptosis and cell death in MCF-7 cells by promoting ROS production, leading to mitochondrial dysfunction. This combination also cleaved the PARP network and activated the caspases. SAL and RSVL synergistically decreased Bcl-2 and downregulated the MAPK pathway by activating p38, increasing the oxidative stress and apoptosis in MCF-7 cells [35]. The combination of RSVL with SAL is also helpful as a novel therapy for TNBC to overcome drug resistance.

## 5. Recent Discoveries and Developments of Natural Products against BC

### 5.1. Novel Verminoside and Their Derivatives

Various adjuvant therapies have been used, including combinations of natural products, in order to improve their susceptibility to BC. These novel products have replaced or limited the use of conventional therapies due to increasing drug resistance. Different natural products have been reported influenced positive effects and could be used as adjuvant therapy by targeting tumors or affecting the EMT process [138]. For example, verminoside (VMS) from *Pseudolysimachion rotundum* interferes with the EMT process by suppressing the invasion and tumor growth; thus, it is widely used for BC treatment [101].

VMS has been used as an anticancer agent and exhibited inflammatory properties. Its anticancer activities have been evaluated in in vivo and in vitro models. Lee et al. [101] carried out an in vivo study in animal models (PyMT/FP635 mouse model) to investigate the anticancer potentials of VMS and showed maximum activity against MDA-MB-231 and MCF7 cells with an IC_50_ value of 10 µM. They found that VMS suppresses epithelial lining growth and the transition of mesenchymal breast cells without activating the ERK signaling pathway (see Table 1). Therefore, VMS can be used as a chemo-adjuvant for the treatment of BC.

VMS is a monoterpenoid that also has been isolated from *K. pinanta*. The biochemical nature of VMS is greatly reflected due to the presence of two hydroxyl groups that are responsible for anticancer and inflammatory properties. It is widely used for inhibiting the EMT process in BC [139]. BC cells under the EMT process have increased mesenchymal characteristics and decreased the formation of epithelial cells and cell linings [109]. These events promoted invasion and migration among BC cells. In the previous studies, it was shown that the excessive use of chemotherapeutic drugs promoted the EMT process. As a result, chemoresistance is one of the major issues tackled through the safe use of natural products as they can inhibit invasion, migration, and EMT in BC development [101].

### 5.2. Novel Phloroglucinol and Derivatives

Phloroglucinols are phenolic compounds that exhibit anticancer and anti-inflammatory activity. Phloroglucinol is the most important novel natural product, and its chemical structure reveals that it has an aromatic ring surrounded by hydroxyl groups. It is composed of two phloroglucinol units linked by a methylene bridge. Calcitrinone A is a type of natural product, novel phloroglucinol, isolated from *C. citrinus*. Calcitrinone A showed potential against MDA-MB-231 BC cells [140].

Calcitrinone A inhibited cell proliferation, invasion, and tumor growth, and stimulated apoptosis in MDA-MB-231 cells. Calcitrinone A is less toxic as compared to other drugs used for the treatment of BC. Calcitrinone A bound with the succinate-coenzyme Q reductase located at complex II of the mitochondrial membrane and blocks their activity. It resulted in the depletion of ATP levels. Calcitrinone A is the most promising anticancer agent used for BC therapy as it interferes with complex II of the mitochondria [141].

An in vivo and in vitro study was conducted by Gaafary et al. [29] to investigate the role of calcitrinone A in the chick chorioallantoic membrane (CAM) and MDA-MB-231 cells, respectively. They found that calcitrinone A interferes with mitochondrial function by blocking succinate coenzyme Q reductase and inhibiting complex II, which can untimely increase ROS production. These events induced apoptosis and cell death in MDA-MB-231 cells (see Table 1). Kim et al. [142] found that phloroglucinol inhibited the epithelial–mesenchymal transition in BC; they also found that phloroglucinol increases ROS production. They found that phloroglucinol also induced apoptosis and cell death in metastatic BC cells.

### 5.3. Role of Viridiflorol against BC

Viridiflorol is a natural organic compound, and its chemical structure contains a cyclopropazulen in its carbon skeleton, responsible for anticancer activity against BC. Viridiflorol, which was isolated from *S. algeriensis*. Furthermore, the Lamiaceae family, including the *Senecio rowleyanus* Jacob, *Mentha aquatica* L, and *Ballota undulata*, are excellent sources of viridiflorol derivatives [143,144]. Previous studies showed that oil secretions from *S. rowleyanus* contained viridiflorol (11%) that possesses anticancer and anti-inflammatory activities [145]. The oil secretions from *Salvia leriifolia* showed anticancer activity against BC (MCF-7 and MBA-MD-231) [146]. Viridiflorol is a class of sesquiterpenoid that isolated various aromatic plants, such as *M. quinquenervia* and *A. edulis*. Extracts of viridiflorol from these plants exhibited anticancer activities against different BC cell lines (MCF-7 and MDA-MB-231) [147]. Essential oil from *Blepharocalyx salicifolius* and *Cyperus longus* is an excellent source of viridiflorol [148,149].

Akiel et al. [27] performed an in vitro study in searching for anticancer activities of viridiflorol against BC MCF-7 cells. The tested viridiflorol showed maximum anticancer activity against MCF-7 with IC_50_ value of 0.1 µM. Memariani et al. [148] conducted in vitro study to investigate the anticancer potentials and apoptosis of viridiflorol. The tested derivatives of viridiflorol showed maximum anticancer activity against MCF7 with IC_50_ value of 31.60% and induced apoptosis at concentrations of 78.23%. Furtado et al. [149] also conducted in vitro study and successfully isolated viridiflorol from *Blepharocalyx salicifolius* that showed anticancer potentials against MDA-MB-231 at concentrations of 46.60 µg/mL. The isolated viridiflorol is also involved in suppressing the cellular metabolism and biological activities of BC cells.

### 5.4. Effect of Vulpinic Acid against BC

Vulpinic acid is a natural product that was isolated from lichens and exhibited anticancer potentials against BC. Lichens secreted a large variety of secondary metabolites effective for the treatment of BC. For example, vulpinic acid is secreted by lichenized fungi which demonstrates anticancer and anti-proliferative activities [150]. Vulpinic acid is a safe and easily assessable metabolite in nature. Vulpinic acid inhibits cell proliferation and invasion, inhibits tumor growth, and stimulates apoptosis in MC-231 cells. Vulpinic acid interacts with the FOXO-3 gene and suppresses their activity, leading to decreased expression of miRNAs [151].

In the previous studies, vulpinic acid has been used for breast treatment as it induces apoptosis and suppresses Bcl-2 [152]. Cansaran-Duman et al. [28] conducted an in vitro study and investigated the role of vulpinic acid on BC by regulating the expression of miRNA levels. They found that vulpinic acid induced apoptosis in MCF-7 by elevating the level of FOXO-3 and Bax, and suppressing Bcl-2 and pro-caspase-3,9, thus altering the tumor suppressor miRNAs (see Table 1). Kilic et al. [153] investigated the anticancer potentials of vulpinic acid against MCF-7, BT-474, MDA-MB-231, and SK-BR-3. They found that vulpinic acid showed anti-proliferative and significant inhibitory effects on the MCF-7. They also found that vulpinic acid induced apoptosis and cell death in MCF-7.

### 5.5. Action of Genistein against BC

Genistein is a naturally occurring compound and possesses anticancer, anti-proliferative, and anti-tumor activities. Fabaceae families are rich in genistein and its derivatives. It is also found in soybeans. It is used to overcome the drug resistance caused by BC and control the reoccurrence of metastatic invasion. It also suppresses the activities of DNA polymerase II [154]. It also arrested the cell cycle at the G2/M phase and induced apoptosis and cell death in MCF-7 and MDA-MB-231 cells. It also inhibited the cell proliferation and progression of BC. Genistein has been used for clinical uses for the prevention of BC because it increases survival rates [102].

Other studies investigated the role of genistein against BC and found that it downregulated CDK-1 and inhibited the expression of Bcl-2, as well as the function of DNA polymerase II. They also found that genistein also increased the expression of p21 and p51 [103,155]. Genistein also showed inhibitory effects on tyrosine kinases and inhibited the cancer progression. Xie et al. [156] investigated that genistein is an effective therapeutic agent that inhibits DNA methylation in MCF-7 and MDA-MB-231 cells by blocking DNA methyltransferase activity. Liu et al. [102] studied that genistein showed inhibitory potentials in the deactivation of IGF-1R and p-Akt. They also found that it decreases the level of Bcl-2 by promoting apoptosis.

## 6. Clinical Trials

Clinical trials focus on the biochemical, pathological, and molecular events for controlling the cancers among the high-risk population. The studies showed that there is a very small number of clinical trials on natural compounds because most of the studies are still investigating in vivo and in vitro studies to check the proper mechanism of action of the natural compounds. However, some clinical trials of natural compounds used for the treatment of BC are under clinical investigation. The details of each clinical trial, i.e., the length of the clinical trial, the clinical phase, the number of participants, and the trial number, are shown in Table 2 [157,158].

## 7. Current Challenges and Future Perspectives

Poor stability and low bioavailability of natural products decrease their therapeutic potential against BC treatment. These challenges could be tackled through a nanotechnology approach by employing the dendrimers to easily and more efficiently improve their delivery for BC therapy. For example, CUR is an excellent source of natural products, whereby nanosuspensions of centrosomes are made for targeting BCs. Further research is needed to improve the functionality of nano-formulated natural products for TNBC [15,161].

Recent challenges for BC treatment in the modern area mainly include drug resistance and high chances of recurrence. For example, it is evident that patients with early BC have a 30% risk of developing metastasis. There is an urgent need to develop novel therapeutics to control the high risk among women [162]. Traditional treatments for the treatment of BC are causing adverse health effects due to increasing drug resistance. For example, there is a high risk of skin cancer in those patients taking BC chemotherapy, which is inevitable. In order to overcome the adverse effects or drug resistance, natural products are reliable sources, as compared to traditional therapies that showed anticancer potentials against BCs [163]. Currently, the synergistic effects of natural compounds have significantly controlled the BC risk. These natural compounds are alternatives to traditional therapies for overcoming drug resistance for BC treatment (see Table 3). However, natural products are also successfully studied in tumor cells of other cancers [164,165,166].

Patients under chemotherapy develop a high risk of chemoresistance caused by ATP-binding cassettes that delay the activity of anticancer drugs [183]. Several natural compounds have been isolated which helped in overcoming the multidrug resistance. For example, β-elemene showed its usefulness against MCF-7 cells [184]. 3,3′-diindolylmethane is also used as an anticancer agent against multidrug resistance. Scientists are also working on the mechanism of action of newly discovered natural compounds that can be used against multi-drug resistance [185].

TNBC has become a global health concern as a limited number of treatments are available. There is a need to isolate novel natural compounds as alternatives for TNBC [186]. For example, CUR and RSVL have been used as effective therapeutic agents for TNBC. These compounds inhibited the proliferation of tumors and induced apoptosis for cell survival [187]. Another prosing natural compound, carnosol, showed the anticancer activity against MDA-MB-231 by arresting the G2 phase of the cell cycle [188].

Several studies showed inconsistencies in the natural products for in vitro and in vivo experimental studies that lower the bioavailability in the experimental models. However, some epidemiological studies on the use of natural compounds for BC have surprising and contradictory effects. For example, soy products can cause serious cellular toxicities and hormonal imbalances [189]. It is necessary to authenticate their anticancer activities, toxicities, and effectiveness prior to using them for BC treatment [76]. Another challenge of using natural compounds is the lack of target specificity, as these compounds have multi-targeted potentials in triggering several signaling pathways rather than specific ones [190].

Oral delivery of natural products remained a major challenge in cancer therapy, and recent attempts have been made in order to improve the oral delivery of ginsengs through nanotechnology. Combining polylactic co-glycolic acid (PLGA) nanoparticles with ginsengs has improved its oral absorption and maximized its potential against MCF-7 cell lines. The nanotechnology approach has been applied to improve the oral absorption of 25-OCH3-PPD via polylactic co-glycolic acid (PLGA). Diameter less than 43 nm showed stability at a pH range of 6.5–7.4, which the FDA has approved for use in therapeutic devices. This approach is helpful for the efficient delivery of poorly soluble natural products that ultimately enhance their use in BC therapy [191]. Another nano-emulsion system coated/fabricated with phospholipids was designed to improve the bioavailability of 25-OCH3-PPD [192]. Cancer stem cells are a major cause of tumor growth initiation and the recurrence of BC due to developing drug resistance. Therefore, targeting the cancer stem cells is crucial for overcoming drug resistance. Natural products, such as therapeutic agents, have become an essential strategy for BC.

Most efficient and reliable therapies are urgently needed for future research of TNBC subtypes for discovering novel biomarkers in order to identify malignant tumor subtypes among high-risk populations. Many natural products from crude extracts have been discovered every year, paving the screening of novel drugs. Many of the plants found in the deep sea are a great source of unknown natural products. This isolation could be a hot spot for the discovery of anticancer-based compounds. Most research carried out for TNBC in vitro studies still lacks in vivo experiments that could explore the mechanism of newly discovered natural compounds [193].

## 8. Conclusions

Recently, attention toward natural products for BC treatment has increased. Natural products have been tested in vivo and in vitro studies for their safe use against BC. Bioactive compounds derived from natural products are the main source of targeting tumor invasion, inhibiting cell-proliferation and cyclins by arresting the cell cycle at M2/S phase, activating caspases, and inducing apoptosis in BC cells. Natural products also showed anticancer and antitumor activities against BC by suppressing NF-κB, PI3K/Akt/mTOR, and MAPK/ERK. Natural products and their respective bioactive compounds act synergistically to overcome drug resistance. Natural products possess low toxicity and are efficiently delivered to the targeted cells; thus, they are helpful for reducing the high risk of BC. The bioavailability issues of natural products can be solved through nanocarriers that are effective for delivering the natural products to the targeted tissues or site. However, several marine plants exhibit a variety of natural products. In the future, screening of more bioactive compounds from marine plants will be helpful to treat and prevent BC. In addition, the efficacy of natural products needs to explore at a clinical level.

## Figures and Tables

**Table 1:** The in vitro and in vivo studies on the role of natural products in downregulating the signaling pathways against various types of BC models.

Extracted Compound	Biochemical Structure	Biochemical Nature	Source	Study Type	BC Type	Animal Model	Key Finding	Mechanism of Action	Reference
VOA	* 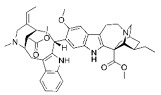 *	Alkaloid	*V. africana*	In vitro	ER-positive, TNBC, and HER2-positive BC	---	Downregulating the PI3K/Akt/mTOR. VOA showed its usefulness against MCF-7 and 4T1 cells with IC_50_ values (0.99, 1.48 μM).	VOA significantly inhibits the phosphorylated AKT and mTOR in BC cells and also decreases the expression of CDK2, cyclin A, E. It also induces apoptosis and cell death in MCF-7 and 4T1 cells by arresting the S phase of the cell cycle.	[34]
Lin A	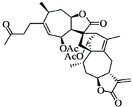	Sesquiterpenoid	*I. lineariifolia*	In vitro	TNBC, and HER2-positive BC	---	Lin A induced apoptosis at a higher concentration of 50% in BC cells (MCF7 and MDA-MB-231 with IC_50_ (4.5 ± 0.3, 7.8 ± 0.6).	Lin A arrests the cell cycle at the G2/M phase, and inhibits cell invasion and cell proliferation in BC cells.	[100,95]
Fisetin	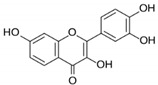	Flavonol	Cucumber, apple, strawberry	In vitro and in vivo	ER-positive, TNBC, and HER2-positive BC	BALB/c mice	Fisetin induced apoptosis in MCF-7, 4T1, and MDA-MB-231 at 40 and 80 μM.	Fisetin acts as an inhibitor of PI3K/Akt/mTOR signaling and inhibits the proliferation and dysregulation of this signaling pathway.	[66]
WG	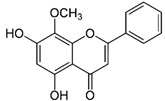	Flavone	*S. baicalensis*	In vitro and in vivo	ER-positive, TNBC, and HER2-positive BC	Chicken chorioallantoic membrane (CAM)	WG showed inhibitory effects on MCF-7 and MDA-MB-231 at 20 and 40 μM.	WG acts as an inhibitor of PI3K/Akt/mTOR signaling and shows inhibition in cell proliferation.	[70]
AP	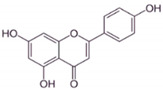	Flavone	*A. cepa*, *C. sinensis*	In vitro	ER-positive, HER2-positive BC	---	It influenced the NF-κB pathway by suppressing the VEGF through deactivating progesterone receptors in BC cells.	It inhibits cell proliferation and migrations by arresting the cell cycle at the G2/M phase. It also suppresses the cyclin A, B, and CDK1 which controls the G2/M phase.	[77]
Oridonin	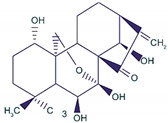	Diterpenoid	*R. rubescens*	In vivo	---	BALB/C athymic nude mice	It induced apoptosis and cell death in BC cells.	Notch 1-4 protein expression is lowered by oridonin therapy, which hinders cancer cell migration and invasion.	[89]
Genistein	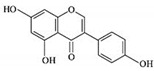	Isoflavones	Soy-based foods	In vitro	ER-positive, TNBC	---	Activation of NF-κB showed potential against MCF-7 at an IC_50_ value of 20 µM.	It inhibits the phosphorylation of IκBα in MCF-7/T47D/MDA-MB-231 cell lines, thus playing a significant role in the regulation of IκBα to the p50.	[73]
GLA	* 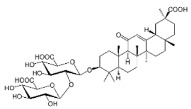 *	Terpenoid	*G. glabra*	In vitro	ER-positive, TNBC, and HER2-positive BC	---	It showed anticancer activity against MDA-MB-231/BT549.	It inhibits invasion and cell proliferation, as well as promote the expression of E-cadherin.	[25]
ATG	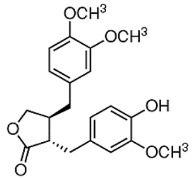	Isoflavones	*S. heteromalla*	In vitro and in vivo	ER-positive, TNBC	BALB/cA-nu	It showed anticancer potentials in MDA-MB-231 cells at 200 μM.	Inhibiting the phosphorylation of MAPK/ERK in MDA-MB-231 cells.	[24]
PPD	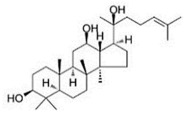	Glycoside	*P. notoginseng*	In vitro and in vivo	TNBC, and HER2-positive BC	BALB/C nude mice	It showed maximum activity below 20 μM against MDA-MB-231.	PPD targets BC cell lines by suppressing the MAPK pathway through the deactivation of ERK1/2, p38, and JNK.	[81]
Kaempferol	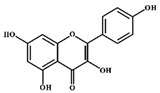	Flavonols	Onions, lettuce	In vitro	ER-positive, TNBC	---	The number of cancerous cells decreased from 85.2% to 50.32% in the G1 phase of the cell cycle. Kaempferol significantly inhibited the BC cells (BT474 and MDA-MB-231) by blocking the critical phases of cell cycles.	Inhibitory actions against different breast cell lines can inhibit the expression of genes involved in MAPK/ERK. This shows that binding with estradiol causes degradation of Erα.	[82]
Cimigenoside	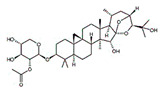	Glycoside	*C. dahurica*	In vitro and in vivo	ER-positive, TNBC	BALB/C nude Crlj mice	Cimigenoside showed maximum anticancer activity against BC cell lines (MDA-MB-231, MCF-7) with IC_50_ (12.6 ± 1.47, 15.6 ± 2.47 μM).	Cimigenoside induces apoptosis in BC cells by arresting the G2/M phase of the cell cycle. An in vitro study of cimigenoside also inhibits/attenuates BC cell proliferation and invasion. An in vivo study inhibited the growth of tumor growth in mice models.	[23,90]
Ginsenosides	* 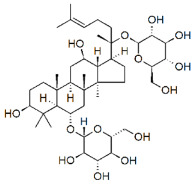 *	Glycosides	*P. notoginseng*	In vitro and in vivo	ER-positive, TNBC	Nu/nu mice	It showed maximum anticancer activity against BC cell lines (MDA-MB-231).	25-OCH3-PPD is involved in arresting the G1 phase of the cell cycle and induces apoptosis in BC cells by downregulating MDM2.	[36]
BA	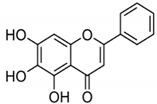	Flavonoid	*Scutellaria baicalensis*	In vitro and in vivo	ER-positive, HER2-positive BC	BALB/c mice	It showed suppression of the NF-κB pathway in the development of human breast epithelial cells (MCF10A).	Suppress the NF-κB signaling pathway, as well as IL-1β, Bcl-2, and VEGF.	[26,96]
VMS	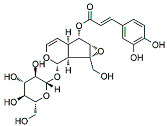	Monoterpenoids	*P. rotundum*	In vitro and in vivo	ER-positive, TNBC, and HER2-positive BC	PyMT/FP635 mouse	It showed maximum activity against MDA-MB-231 and MCF7 cells with IC_50_ value (10 µM).	VMS suppresses the growth of epithelial lining and the transition of mesenchymal breast cells.	[101]
Calcitrinone A	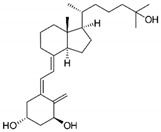	Phloroglucinol	*C. citrinus*	In vivo and in vitro	ER-positive	Chick chorioallantoic membrane (CAM)	Calcitrinone A induced apoptosis and cell death in MDA-MB-231 cells.	Calcitrinone A interferes with mitochondrial function by blocking succinate coenzyme Q reductase and ultimately inhibits the complex II that increases the production of ROS.	[29]
Vulpinic acid	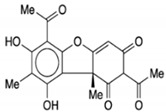	Butenolide	Lichens	In vitro	ER-positive, TNBC, and HER2-positive BC	---	Vulpinic acid induced apoptosis in MCF-7.	Elevate the levels of FOXO-3 and Bax, and suppress the expression of Bcl-2 and procaspase-3/9 to enhance the activity of tumor suppressor miRNAs.	[28]
Genistein	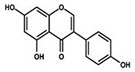	Isoflavone	Fabaceae family	In vitro	TNBC, and HER2-positive BC	---	It induced apoptosis and cell death in MCF-7 and MDA-MB-231 cells. It also inhibited cell proliferation and progression in BC.	Arresting the cell cycle at G2/M phase, downregulating CDK-1, and inhibiting the expression of Bcl-2 and the function of DNA polymerase II.	[102,103]
CUR + BBR	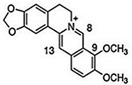	Diarylheptanoid, isoquinoline alkaloid	*Curcuma longa*, berberine from *Rhizoma coptidis*	In vitro	ER-positive, TNBC, and HER2-positive BC	---	It showed effects against BC cell lines (MDA-MB-231 and MDA-MB-468) at *p* ≤ 0.010.	The EMT process in the case of BC is impaired.	[104,105]
BA + 5-FU	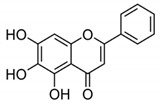	Flavonoid (BA)	*Scutellaria baicalensis*	In vivo	---	Swiss albino mice	It showed inflammation by inhibiting the VEGF, IL-1β, and NF-κB.	Inflammation is inhibited by the VEGF, IL-1β, and NF-κB, which play significant roles in preventing BC.	[106]
MG + 5-FU	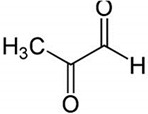	Polyphenolic (MG)	Coffee, wine	In vivo and in vitro	ER-positive, TNBC, and HER2-positive BC	BALB/c mice and Swiss albino mice	It showed anticancer activity against the BC cell line (MCF-7).	Arresting the cell cycle at MG G0/G1 phase induces apoptosis and cell death by increasing Bac-2 and caspase 9.	[30]
RSVL + SAL	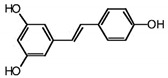	Polyphenol	Red grapes	In vivo and in vitro	ER-positive, TNBC, and HER2-positive BC	Swiss albino mice	It showed anticancer activity against MDA-MB-231.	Arresting the S1 phase of the cell cycle also induces apoptosis in BC cells. Other activities include inhibition and cell proliferation. It acts as an antioxidant by preventing the DNA dame and suppressing the tumor growth. SAL inhibits the epithelial mesenchymal transition, and suppresses p53, COX-2, and Beclin.	[107,108]

Note: VOA: voacamine; Lin A: lineariifolianoid A; ATG: arctigenin; WG: wogonin; AP: apigenin; GLA: glycyrrhizin; PPD: protopanaxadiol; BA: baicalin; VMS: verminoside; CUR: curcumin; 5-FU: 5-fluorouracil; BBR: berberine; RSVL: resveratrol; SAL: salinomycin; MG: methylglyoxal; TNBC: triple-negative breast cancer; ER: estrogen receptor.

**Table 2:** The representation of clinical trial studies of some natural compounds against BC.

Natural Compound	Nature	NCT No.	Number of Participants	Disease Type	Dose/Concentration	Duration of Trial	Trial Phase	Study Location	Reference
Curcumin	Polyphenol	NCT01740323	30	BC	8 g	8 weeks	Phase II	USA	www.clinicaltrials.gov (accessed on 27 April 2022)
Resveratrol	Stilbenoid	---	39	Metastatic BC	5 or 50 mg	3 months	Phase I	USA	[157,158]
Berberine	Alkaloids	NCT03281096	1000	Invasive BC, colorectal	300 mg	4 weeks	Phase II and III	China	www.clinicaltrials.gov (accessed on 27 April 2022)
Curcumin (iv) + Paclitaxel	Polyphenol	NCT03072992	75	Metastatic BC	300 mg	12 weeks	Phase II	Armenia	www.clinicaltrials.gov (accessed on 27 April 2022)
Quercetin	Carotenoids	---	42	Advanced BC	200 mg	2 weeks	Randomized crossover clinical trial	UK	[159,160]
Resveratrol	Stilbenoid	NCT04266353	50	TNBC	150 mg	2–4 weeks	Suspended (due to COVID-19)	California	www.clinicaltrials.gov (accessed on 27 April 2022)
Curcumin	Polyphenol	NCT01975363	30	BC (obese women)	100 mg	3 months	Pilot trial	USA	www.clinicaltrials.gov (accessed on 27 April 2022)
Genistein	Isoflavon	NCT00099008	30	BC	10 or 20 mg	84 Days	Completed	US	www.clinicaltrials.gov (accessed on 27 April 2022)

**Table 3:** The natural compounds that overcome drug resistance in BC.

Natural Compound	Nature	Dose/Concentration	Target	Cell Mode	Chemo Drug	IDS (x-Fold)	Reference
Ginsenosides	Glycosides	40 µM	MCF	ADM	Doxorubicin	29.2	[167,168]
Baicalin	Flavonoid	150 µg/mL	MDR1 and MRP1	MCF7/ADR	Doxorubicin	6.5	[169,170]
Quercetin	Carotenoids	50 µM	MDR1 and MRP1	MCF7/ADM	Cisplatin	3.5	[171,172,173]
Berberine	Alkaloids	20 µM	MDR1	MDR1	Vincristine	3.2	[174,175]
Ginsenoside Rb1	Glycosides	80 µM	MDR1	MCF-7/ADR	Vincristine	2.5	[176,177]
Apigenin	Flavone	13 µM	MDR1	HCT	5-FU	4.9	[169,173]
Curcumin	Alkaloids	25 µM	MDR1	Various	Various	4.5	[178,179]
Oridonin	Diterpenoid	3 µM	MDR1	MCF7/ADM	Various	8.5	[180,181]
Ginsenoside Rg3	Glycosides	30 µg/mL	MDR1 and MRP1	MCF7/ADR	Various	8.5	[182]

Note: HCT: hematocrit; ADR: adverse drug reactions; MCF-7: Michigan Cancer Foundation 7; IDS: increase in drug sensitivity; MDR1: multidrug resistance 1; MRP1: multidrug resistance protein 1.

## Data Availability

Not applicable.

## Abbreviations

**Abbreviation**	**Explanation**
ADAM-10	A disintegrin and metalloproteinase domain-containing protein 10
ADR	Adverse drug reactions
Akt	Protein kinase B
AP	Apigenin
BA	Baicalin
Bad	Bcl-2 antagonist of cell death
Bax	Bcl-2-associated X protein
BBR	Berberine; BC: breast cancer
Bcl-2	B-cell lymphoma 2
CDK	Cyclin-dependent kinase
Chk	Checkpoint kinase
CUR	Curcumin
DATS	Diallyl trisulfide
EGCG	Epigallocatechin-3-gallate
EGF(R)	Epidermal growth factor (receptor)
ER	Estrogen receptor
ERK	Extracellular signal-regulated kinase
5-FU	5-Fluorouracil
HCT	Hematocrit
IKK	IκB kinase
IL-1β	Interleukin 1 beta
IDS	Increase in drug sensitivity
JNK	C-Jun N-terminal kinase
Lin A	Lineariifolianoid A
MAML	Mastermind-like protein
MDM2	Mouse double minute 2 homolog
MEK	Mitogen-activated protein kinase kinase
MCF-7	Michigan Cancer Foundation 7
MDR1	Multidrug resistance 1
MG	Methylglyoxal
MMP	Matrix metalloprotein
mTOR	Mammalian target of rapamycin
mTORC-2	mTOR Complex 2
MRP1	Multidrug resistance protein 1
NFAT1	Nuclear factor of activated T cells 1
NF-κB	Nuclear factor kappa B
NICD	Notch receptor intracellular domain
p53	Inducible gene 3
PARP	Poly (ADP-ribose) polymerase
PDK1/2	3-phosphoinositide dependent kinase-1/2
PI3K	Phosphatidylinositol 3-kinase
PIP-3	Phosphatidylinositol (3,4,5)-trisphosphate
PPD	Protopanaxadiol
PTEN	Phosphatase and tensin homolog
RBP-Jκ	Recombinant signal binding protein for Immunoglobulin kappa J region
RSVL	Resveratrol
SAL	Salinomycin
TNBC	Triple-negative breast cancer
VEGF	Vascular endothelial growth factor
VOA	Voacamine
VS	Verminoside
WG	Wogonin
